# Dynamics of Plant Metabolism during Cold Acclimation

**DOI:** 10.3390/ijms20215411

**Published:** 2019-10-30

**Authors:** Lisa Fürtauer, Jakob Weiszmann, Wolfram Weckwerth, Thomas Nägele

**Affiliations:** 1 Plant Evolutionary Cell Biology, Department Biology I, Ludwig-Maximilians-Universität München, 82152 Planegg-Martinsried, Bavaria, Germany; lisa.fuertauer@lmu.de; 2 Department of Ecogenomics and Systems Biology, University of Vienna, Vienna 1090, Austria; jakob.weiszmann@univie.ac.at (J.W.); wolfram.weckwerth@univie.ac.at (W.W.); 3 Vienna Metabolomics Center, University of Vienna, Vienna 1090, Austria

**Keywords:** cold acclimation, metabolic reprogramming, carbohydrates, subcellular metabolism, sucrose cycling, enzyme activity, Arrhenius equation, kinetic modelling, *Arabidopsis thaliana*

## Abstract

Plants have evolved strategies to tightly regulate metabolism during acclimation to a changing environment. Low temperature significantly constrains distribution, growth and yield of many temperate plant species. Exposing plants to low but non-freezing temperature induces a multigenic processes termed cold acclimation, which eventually results in an increased freezing tolerance. Cold acclimation comprises reprogramming of the transcriptome, proteome and metabolome and affects communication and signaling between subcellular organelles. Carbohydrates play a central role in this metabolic reprogramming. This review summarizes current knowledge about the role of carbohydrate metabolism in plant cold acclimation with a focus on subcellular metabolic reprogramming, its thermodynamic constraints under low temperature and mathematical modelling of metabolism.

## 1. Introduction

Due to their sessile lifestyle, plants have to cope with a changing environment. Fast acclimation to unfavourable surroundings is necessary to ensure survival. The responses of plants to abiotic stress, e.g., cold, heat or drought, are diverse and highly dynamic. Exposing plants to abiotic stress induces an immediate response before plants acclimate to establish a new metabolic homeostasis [1]. Range boundaries of many temperate herbaceous plants, and also of *Arabidopsis thaliana* are defined by low temperature [2]. It is estimated that only ~5% of the earth’s surface is frost free, which immediately implies that frost has a significant impact on agricultural production [3]. In many temperate plant species, the exposure to low, non-freezing temperatures results in an increase of freezing tolerance through a multigenic process termed cold acclimation. After the immediate stress response, which is characterized by short-term and transient physiological, biochemical and molecular processes, metabolism is stabilized to a new homeostasis [4]. Plant acclimation to low temperature has been of interest for a long time [5] and continues to be of relevance with respect to the modern challenges of climate change, which lead to greater local temperature extremes and locally increased risk of frost damage in spring [6]. Environmental factors differ across a species range. For example, a comparison of Swedish *Arabidopsis thaliana* accessions showed that the most tolerant ones have historically experienced more precipitation combined with low temperature, and that this abiotic factor combination seems to play a crucial role in increasing freezing tolerance [7].

The process of cold acclimation is induced after a short time period of exposure to low but non-freezing temperatures [8]. It plays an important role in plant evolution and ecology, as indicated by the high mortality at -8 °C of non-acclimated plants compared to acclimated plants which has been described for more than 70 natural *Arabidopsis* accessions [9]. The adverse effects of low temperature are counteracted during the phase of cold acclimation by extensive reprogramming of transcriptome, proteome and metabolome. There is no unique metabolite, transcript or pathway that can be assigned as responsible for cold tolerance; rather, a multifaceted reorganisation of metabolic homeostasis seems to be necessary [10]. Across the plant kingdom, diverse strategies are employed to react to low temperature, and thus, although there are certain conserved mechanisms it remains difficult, if possible at all, to define a universally valid cold response model [11]. Even within the well-studied model plant *Arabidopsis thaliana,* genetic architecture varies across the species range [7], which also contributes to the strong variation in intraspecific cold response, cold hardiness and freezing tolerance [12,13]. Nevertheless, finding common mechanisms as well as understanding specialized responses will help breeders to establish strategies to stabilize crop yields with respect to potential frost risks. This review provides an overview of the role of carbohydrates and the regulation of their metabolism during plant cold acclimation with a focus on metabolic reprogramming, subcellular metabolism and mathematical modelling to study biochemical regulation under low temperature.

## 2. Perception of Low Temperature

Cellular membrane systems are primary targets for freezing injury due to extracellular ice formation and cellular desiccation [14,15]. Extracellular ice formation leads to water loss in cells and causes cellular dehydration and cell shrinkage, thus affecting the integrity of cellular membrane systems [16,17]. The change in membrane fluidity is recognised as a signal of cold perception within cells [18], as fluidity changes can lead to, e.g., an opening of Ca^2+^ channels [19,20]. Modified Ca^2+^ concentrations induce signalling cascades, e.g., through mitogen-activated protein kinases (MAPKs), eventually triggering comprehensive changes on the transcriptomic level [19,21]. Cold also activates other plasma membrane located kinases, e.g., responsive protein kinase 1 (CRPK1), which were found to transduce signals into the nucleus and possibly affect C-repeat Binding Factor (CBF) signalling [22]. Furthermore, decreased temperature, and thereby decreased membrane fluidity has been found to activate a diacylglycerol kinase (DAGK) pathway which is thought to prevent membrane damage under freezing conditions by its metabolic products [18,23].

Transcriptional regulation plays an essential role in cold acclimation. The CBF pathway in *Arabidopsis* is a central player in freezing tolerance, which illustrates the complexity of the cold acclimation process [24]. The CBF locus and its three genes *CBF1-3* encode transcription factors (TFs) that are induced within minutes of exposure to low non-freezing temperatures [25]. CBF TFs alter the expression of more than 100 cold-regulated (*COR*) genes, also known as the CBF regulon [26,27]. Finally, the coordinated response of *COR* genes contributes to higher survival rates of plants under freezing stress [28]. Natural accessions of *Arabidopsis thaliana* also show differential regulation of the CBF pathway, which correlates with the variation in freezing tolerance and emphasizes the role of the CBF pathway as an evolutionary factor [29]. The CBF pathway itself is tightly regulated by other transcription factors, phytohormones, Ca^2+^ binding receptor kinases, the circadian clock, light intensity, light quality and by post-translational modifications (PTMs) [30,31,32]. Carbohydrates are also involved in regulation of several *COR* genes. For example, sucrose was found to enhance transcription of *COR78* [33], and also targets like galactinol synthase seem to be directly controlled by the CBF regulon [34]. Further, CBF1 modulates accumulation of DELLA proteins [35,36] and sucrose potentially stabilizes DELLAs [37]. The analysis of freezing tolerance across a diverse set of natural accessions of *Arabidopsis thaliana* revealed a strong correlation between freezing tolerance and MYB transcription factors including production of anthocyanin pigment 1 and 2 (PAP1 and 2), which are involved in the regulation of flavonoid metabolism [12]. A functional role of PAP1 and 2 in *Arabidopsis* cold acclimation and freezing tolerance was discussed recently [38], which might be related to the suggestion that DELLAs activate anthocyanin biosynthesis by stimulation of PAP1/MYB75 transcription factors [37].

Cold acclimation significantly affects cellular protein amount. Thus, focusing solely on changes in transcript levels is often not enough to reveal the actual state of metabolism. Post-transcriptional mechanisms, post-translational modifications and differential regulation of protein isoforms are crucially involved in cold stress response [39]. Proteome and enzyme activity analysis together with metabolome analysis are necessary due to their reciprocal interaction with transcriptional and translational regulation. Moreover, modification of gene expression at the transcript level frequently does not correlate with protein level [40], and similarly, at low temperatures enzyme activities often do not correlate with the dynamics of protein amount. In general, low temperature has a wide-ranging effect on photosynthesis, carbohydrate metabolism, polyamine synthesis, reactive oxygen species (ROS) scavenging, protein folding, stabilizing cell structure and cell membrane integrity [39,41]. Frequently, involved proteins are significantly affected by the CBF regulon [42]. For example, abundance and phosphorylation of COR 78 protein was found to positively correlate with the acclimation state of natural *Arabidopsis* accessions and their carbohydrate accumulation capacity [43]. Increased abundance of COR78 and COR15B is a typical consequence of cold exposure, which has also been observed in metabolic mutants with enzymatic deficiency in starch or sucrose metabolism [44]. This observation indicates that although carbohydrates are essentially involved in cold acclimation, the study of their regulatory role is complicated by such functional redundancy and the robustness of underlying regulatory networks.

## 3. Tight Regulation of Photosynthesis and Carbohydrate Metabolism Prevents ROS Generation under Low Temperature

If temperature drops, photosynthetic light reactions and the central carbohydrate metabolism need to be immediately reprogrammed to prevent any imbalances that would cause production of ROS, cell damage or cell death [4,45]. Changing temperature immediately affects photosynthesis [46]. Low temperature might induce a reduction in the size of PSII associated antenna [4]. Within the first minutes of cold exposure, plants compensate for the high PSII excitation pressure either by diverting energy from PSII to PSI by state transition or by dissipating heat via non-photochemical quenching [4]. The photosynthetic rate is not only controlled by the amount of associated proteins, but also fine-tuned by the specific production of isoforms with optimized performance or adaptions in the activation state according to the prevalent temperature [47,48]. An initial decrease in photosynthetic rate is not only due to direct effects in the photosynthetic apparatus, as systemic thermodynamic effects influence the enzyme activities of the overall metabolism. For example, the electron transport chain in chloroplasts relies on a continuous supply of NADP^+^ as an electron acceptor, which is mainly provided by usage of NADPH + H^+^ in carbon fixation reactions. A decreased activity of Calvin cycle enzymes has recently been found to possibly contribute to over-reduction-associated damage to the photosystem and inhibition of the photosynthetic rate [49].

The continuous function of photosynthesis and the Calvin cycle relies on the exchange of triose phosphate (TP) from the chloroplast with orthophosphate (Pi) from the cytosol via the triose phosphate/phosphate translocator (TPT), which directly links photosynthetic processes to the energy balance and carbohydrate metabolism in the cytosol [50,51]. Enzymatic sucrose biosynthesis via sucrose phosphate synthase (SPS) significantly affects Pi concentration in the cytosol, and it has been indicated that limitations in SPS capacity disturbs the export of triose phosphate from the chloroplast [50]. If triose phosphates cannot be exported in sufficient quantities and starch metabolism cannot compensate for the excess amount of triose phosphates, photosynthesis may run into disequilibrium, resulting in ROS production [52]. In this context, increased cold tolerance in *Arabidopsis* was linked to a higher capacity for sucrose biosynthesis, which prevents a bottleneck in metabolism under cold exposure [53,54]. The accumulation of TP and the resulting Pi limitation in the chloroplast might further lead to a strong inhibition of photosynthesis by damaging the photosystem through over-reduction of the electron transport chain [55]. This could be relieved by direct supplementation with Pi [56]. Product inhibition might intensify a decrease in the enzyme activity of carbon fixation and sucrose biosynthesis due to affected metabolic sink activity and decreased assimilate export [57,58]. It has been suggested that regulation of the photosynthetic apparatus via redox regulatory networks is crucial for chilling stress acclimation [59]. A possible link between chloroplast antioxidant capacity and carbohydrate availability was shown in *Arabidopsis*. Transcripts of genes encoding for the photosynthetic electron transport chain and chloroplastidic antioxidant enzymes were decreased upon external sucrose feeding [60]. Nevertheless, through acclimation the optimum temperature for photosynthesis can be shifted and rates of photosynthesis can be reached which are similar to those under ambient temperature [61].

## 4. Carbohydrate Dynamics of Plant Cold Acclimation

Carbohydrates are the primary products of photosynthesis, and they play a central role in energy metabolism, developmental processes, stress signalling and temperature acclimation. Reprogramming of primary metabolism during cold acclimation typically results in the accumulation of soluble sugars, sugar alcohols, organic acids, amino acids, polyamines [62] and substrates for secondary metabolites [63]. This accumulation eventually allows plants to withstand lower temperatures when compared to non-acclimated plants [13]. Sugars play diverse roles which are, e.g., stabilization of membranes, osmo-protection, and protection of proteins from desiccation. Further, sugar accumulation might also result from reduced sink activity because growth retardation at low temperatures is stronger than the reduction of photosynthetic activity [4]. In general, reactions in primary metabolism are tightly regulated and closely linked to the circadian clock to ensure continuous carbohydrate availability [64,65]. Clock components are significantly influenced by low temperature [66] and sugars are also known to be important for entrainment of the clock [67]. Besides the clock components, light itself is deemed to be essential for cold acclimation [68,69].

It has been suggested that carbohydrates directly influence cell membrane stability by interacting with the membrane interface and therefore support the maintenance of membrane integrity under freezing conditions [70,71]. For example, sucrose can interact with the phosphate in lipid headgroups, thereby decreasing membrane permeability [72]. Carbohydrates have been found to stabilize in vitro liposomes against leakage of aqueous content, which suggests a cryoprotective role in vivo [15]. Fructans have been found to move via vesicle transport from vacuoles to the apoplast where they can assist in stabilizing the plasma membrane [73]. Further, sugar transport proteins are believed to play a role in vacuolar fructan export [65]. Raffinose family oligosaccharides (RFOs) are known to protect membranes under cold stress and contribute to higher freezing resistance [74]. Raffinose is synthesised within the cytosol and transported into plastids to protect thylakoid membranes, contributing to PSII integrity and acting as a potential ROS scavenger [75,76,77]. Furthermore, hydrogen bonds between sugars and proteins are discussed to inhibit dehydration-induced protein unfolding [78].

Starch is a direct product of photosynthesis and a storage compound for carbon. Starch biosynthesis and breakdown are tightly regulated during abiotic stress. Many enzymes involved in starch metabolism are redox regulated [79,80]. During cold stress, starch metabolism in *Arabidopsis* has great flexibility in the way it reacts to differences in growth conditions and it is a determinant of plant fitness under abiotic stress [81]. Starch degradation is an initial response [82] as starch metabolism has the potential to relieve product inhibition effects on Calvin cycle-associated enzymes and might allow the release of Pi in chloroplasts under cold. Increased activity of beta-amylases supplements maltose accumulation during cold exposure [83,84]. Further, mobilisation of starch seems to differ between different natural accessions of *Arabidopsis* and might influence their cold acclimation capacities [43]. Interestingly, impairment of plastidial α-glucan phosphorylase resulted in no significant changes in the starch content of *Arabidopsis* leaves, but reduced survival under stress [85]. This highlights that the dynamics of synthesis and breakdown pathways rather than the absolute amount of starch might be responsible for metabolic reprogramming and survival under abiotic stress. Starch has several roles in both source and sink tissues and starch degradation into sugars has a pivotal role for plant cold stress responses via offering osmo-protective sugars and rapid energy supplies [86].

Maltose, a product of starch degradation, might serve as a direct osmo-protectant in chloroplasts [83] by protecting stromal proteins from dehydration [86]. Maltose supplies biosynthesis of other carbohydrates like hexoses and raffinose, but also proline [82,84] to fuel and maintain carbon metabolism. A direct correlation between freezing tolerance in *Arabidopsis* accessions and the degree of accumulation of raffinose and proline has been observed [87]. Proline is well-known to accumulate during stress response, affecting signaling events, cryoprotection and redox balance in several plant species [88,89]. In a protein-protein interaction network, delta 1-pyrroline-5-carboxylate synthase 2 (P5CS2), which is a central enzyme in proline biosynthesis, indicated a linkage to heat-shock proteins and to the interface of primary and secondary metabolism [44]. In general, gene expression related to secondary metabolism is well correlated with freezing tolerance [12,90]. In *Arabidopsis*, biosynthesis of secondary metabolites, e.g., flavonoids, is induced during cold exposure [91,92]. The substance class of flavonoids is estimated to comprise more than 8000 metabolites and flavonoid metabolism may comprise 20% of the total carbon flux in a plant cell [93,94]. Flavonoids frequently contain sugars like rhamnose, arabinose, glucose, galactose [95,96], which directly shows the necessity of a regulatory and metabolic interaction between plant carbohydrates and secondary metabolism during cold acclimation. Several flavonoid biosynthesis mutants with reduced flavonoid content showed impaired freezing tolerance, and the contribution of flavonoids to freezing tolerance was shown to be genotype-dependent [38]. Flavonoid metabolism was found to be regulated via post-transcriptional mechanisms as the corresponding transcripts and metabolites correlated poorly in response to cold [97].

## 5. Subcellular Metabolic Regulation during Cold Acclimation

Cell organelles and compartments are interconnected by various transport and shuttle systems that enable a regulated exchange of metabolites across biological membrane systems [98,99]. Analyses of crude whole cell extracts of metabolites and proteins are suitable to record the overall stress response of metabolism. Nevertheless, information about organelle specific subcellular alteration of biosynthetic pathways is strongly limited by analysis at the whole cell level. As a result of analysis at a whole cell level, the functions of metabolites and proteins might be hidden or overlooked, which can lead to misinterpretation of results. Specific changes in subcellular concentrations of potential stress protectants can have a massive influence on successful stress responses. Hence, a combination of subcellular metabolite information with subcellular transport activity, e.g., by Tonoplast Sugar Transporters (TSTs) [100,101], Sugars Will Eventually Be Exported Transporters (SWEETs) [102], or plastidic Sugar Transporter (pSUT) [103], essentially supports the identification of regulatory strategies involved in cold acclimation.

Applying the method of nonaqueous fractionation (NAF) makes it possible to determine metabolites from one sample at a subcellular level, as it reveals chloroplastic, cytosolic, vacuolar and mitochondrial information [104,105,106]. NAF has been applied in several studies to investigate subcellular metabolism under cold exposure. Knaupp and colleagues found indications for the stabilization of photosystem II by plastidial raffinose [77]. Leaves developed in the cold showed lowered cytosolic pyruvate and 3-phosphoglycerate levels, but increased dark respiration compared to cold shifted leaves [107]. It was discussed that either the reprogrammed metabolism has higher maintenance costs, or this might be a precautionary effect due to environmental changes that require rapid reorganization of metabolism [107]. Analysis of natural accessions of *Arabidopsis thaliana* indicated distinct mechanisms of carbohydrate reallocation between different freezing-tolerant accessions [108]. Further analysis indicated that a freezing sensitive accession enhanced its subcellular redistribution of metabolites between subcellular compartments during acclimation whereas a freezing tolerant accession was found to intensify the accumulation of sugars and amino acids [106]. The reprogramming of plastidial primary metabolism was found to be important to prepare for continuation of growth under low temperature [109], and the hexokinase 1 deficient *Arabidopsis* mutant *gin2-1* showed a delayed accumulation of protective plastidial metabolites, like proline in response to cold treatment [110]. These examples provide strong evidence of the suitability of subcellular fractionation to reveal the regulatory mechanisms and dynamics of metabolism under low temperature.

## 6. Sucrose Cycling—Stabilization of Metabolism in a Changing Environment

Subcellular cycling, i.e., cyclic biosynthesis and degradation of sucrose was shown to be significantly influenced by low temperatures and is thought to play an essential role in stabilizing photosynthesis during environmental changes [111,112]. In leaf mesophyll cells, sucrose is synthesized in the cytosol by a sequential reaction of SPS and sucrose phosphate phosphatase (SPP). UDP-glucose and fructose-6-phosphate are substrates for SPS to synthesize sucrose-6-phosphate whereas SPP releases Pi yielding sucrose [113].

Sucrose biosynthesis is a central part of energy metabolism and was shown to be a limiting factor in cold acclimation [53,54,114]. Overexpression lines of SPS showed an improved photosynthetic performance and increased freezing tolerance after cold acclimation [53]. The regulation of SPS activity is multi-layered and comprises protein phosphorylation, which inactivates SPS [115]. Additionally, its activity is stimulated by glucose-6 phosphate and inhibited by UDP and Pi [116,117]. Invertases (Inv) catalyse the hydrolytic cleavage of sucrose to glucose and fructose. Invertases are located in several compartments comprising cytosol, vacuole, mitochondria, chloroplast and the cell wall [113,118,119]. From an evolutionary perspective, it is hypothesized that different invertases have evolved for different functions, e.g., coevolution of cell wall invertases and vascular tissue [119]. Particularly in sink tissues, sucrose can also be cleaved by sucrose synthase (SuSy) to form fructose and UDP-glucose or ADP-glucose [120]. Glucose and fructose are re-phosphorylated by hexokinases to yield hexose phosphates, which are again substrate for sucrose biosynthesis.

Continuous sucrose breakdown and re-synthesis appears to be energetically wasteful (futile cycle), but this allows precise control over carbohydrate partitioning [113]. For cotyledons and leaves of various species and experimental setups, sucrose recycling flux was estimated to account for 10–30% [121,122,123]. Stability analysis of kinetic parameters indicated that hexokinase is an important regulator of the cycle, while sucrose degradation by invertases appeared to be secondary [121,124]. By confirming the limitation of sucrose cycling via hexokinase, it was shown that a deficiency in glucokinase activity resulted in sucrose accumulation and enhanced root respiration [125]. Additionally, impairment of hexokinase 1 in the *gin2-1* mutant might indicate problems in assimilate transport and shoot growth [126]. Conversely, the strong stimulation of sucrose cycling was observed in detached cotyledons in *Ricinus communis* [122]. In addition to cytosolic sucrose cycling and export to phloem, another cyclic reaction across the tonoplast has been suggested to play a role in stabilizing metabolism due to environmental cues. Sucrose can be cleaved by vacuolar invertases, yielding hexoses, which can be transported into the cytosol to fuel the cytosolic hexose pool [112].

The simulation of effects of fluctuating environmental conditions on primary carbohydrate metabolism, considering millions of possible enzyme kinetic parameters, has allowed analysis of the stability behaviour of the system [111]. Analysis revealed a non-intuitive link between vacuolar and plastidial metabolism, as a perturbation of vacuolar sucrose and hexose metabolism interfered with the regulation and stabilization of plastidial and cytosolic carbohydrate metabolism and photosynthetic performance [111]. Supporting this theory, kinetic modelling of carbon metabolism under cold stress, which compared a freezing tolerant and a freezing sensitive natural accession of *Arabidopsis thaliana,* revealed different strategies for partitioning sucrose cleavage via cytosolic/neutral or vacuolar/acidic invertase. The freezing tolerant accession shifted sucrose cleavage capacity from the cytosol into the vacuole whereas the freezing sensitive accession maintained a high rate of cytosolic sucrose cleavage [112]. Deficiency in vacuolar sucrose cleavage capacity lead to a disturbed cytosolic hexose metabolism, an affected ADP/ATP ratio, and finally lead to decreased photosynthetic CO_2_ uptake under cold and high light stress conditions [112]. The central role of invertases during early stress conditions, e.g., drought and water stress, was also shown to play a crucial role in maize leaves [127,128,129].

In conclusion, those examples show that sucrose cycling not only allows precise control over carbohydrate partitioning, it also serves as an energy balancing mechanism that reacts efficiently to sudden environmental changes. Subcellular information is not only necessary to unravel the metabolic pathway regulation, but also for biotechnological applications, e.g., metabolic engineering [130] and to feed mathematical models of plant metabolism in order to quantify non intuitive dynamics of metabolic systems [131].

## 7. Mathematical Modelling at Low Temperature: Kinetics and Thermodynamic Constraints

Experimental analysis of dynamics in metabolism via *omics* techniques has significantly advanced our knowledge and understanding of plant cold acclimation. Recorded transcript abundance, protein levels, metabolite concentrations and enzyme activities enable the simultaneous elucidation of pathway regulation. For the analysis of such data sets, regression and correlation analysis are widely used to characterize system dynamics. However, analysis of plant response to temperature also needs to consider non-linear system dynamics due to thermodynamic constraints [132]. Numerous, often unknown, regulatory effectors like feedback/feedforward loops significantly affect metabolic reprogramming. A combination of multivariate statistics, mathematical modelling, and pattern recognition yielded promising predictive information on the biochemical regulation of plant metabolism [133]. The availability of genome-scale metabolic reconstructions of plant metabolism has supported the functional integration of experimental high-throughput data, which has played a crucial role in predicting observed phenotypes [134,135]. Furthermore, a combined sink-source model of plant metabolism essentially supports crop engineering and emphasizes the essential role of mathematical modelling [130]. Thus, it can be expected that future *in silico* concepts of analyzing plant metabolism will crucially support functional data integration from the genome to the ecosystem scale [136].

Many mathematical models of plant metabolism consist of ODEs (ordinary differential equations) that describe time dependent changes, e.g., of metabolite concentrations, by the sum of synthesizing and degrading reaction rates. Various parameters like enzyme abundance, post-translational modification, and thermodynamic constraints as well as inhibitor and activator concentrations define these reaction rates [131,133]. To study plant metabolism, the most commonly used modelling approaches are constraint-based modelling (CBM) and kinetic modelling. CBM is applied to large networks and compares the steady-state behavior of different conditions, while kinetic modelling is the method of choice to elucidate dynamic system behavior [1,137]. In general, kinetic models tend to consider only a relatively low number of reactions because of experimental limitations in recording enzyme kinetics and activities. Consequently, kinetic model construction comprises critical steps for metabolic network simplification and various assumptions about the comparability of in vivo and in vitro measurements [138]. Despite such critical assumptions, mathematical modelling has been proven to efficiently support the analysis of plant-environment interactions. For example, Calvin-Benson cycle enzyme activity was shown to be affected by metabolite concentrations outside of the chloroplast [139]. Further, ODE model simulation and mathematical analysis have revealed diurnal pathway regulation of plant metabolism [140], diurnal and circadian sensors [141] and critical temperatures for sucrose biosynthesis [54]. Mathematical modelling has supported the analysis of subcellular sugar metabolism during cold exposure [108,112], and sink-source dynamics [142]. Finally, although kinetic modeling frequently comprises strong network simplification, it represents a powerful strategy to reveal and predict temperature-induced metabolic reprogramming.

Changes in the environmental temperature regime have an immediate effect on enzymatic activities and reaction rates following thermodynamic laws. According to the van’t Hoff rule, the velocity of enzymatically catalysed reactions decreases by a factor 2–3 per each 10 °C reduction [143]. This theory was developed further as the so-called Arrhenius equation [144,145]. The Arrhenius equation (Equation 1) is a simple, yet precise way to investigate temperature dependent changes in enzyme reaction velocities in biological systems [146], nevertheless, there are limits due to thermal stability of proteins [147]. It describes the rate constant k of a chemical reaction as the product of a constant C and an exponential term (Equation 1). The constant C comprises information about collision frequency and geometric molecule positions. The unit of C varies depending on the reaction order, and for a first order reaction it is denoted by [s^−1^]. The exponential term comprises the activation energy E_A_ [J mol^−1^], temperature T [K] and the universal gas constant R [J K^−1^ mol^−1^].

k = C * exp(-E_A_ R^−1^ T^−1^) (1)

In biochemical reactions, E_A_ frequently ranges between 40 and 50 kJ mol^−1^ and comprises various steps of catalytic activity [148]. To yield an approximate value for in vivo enzyme activity and flux estimation under low temperature, maximal enzyme activity might be experimentally determined by applying the plant growth temperature, e.g., 5 °C, for enzyme activity measurements. Yet, this frequently results in a complicated experimental setup, which might also affect the statistical robustness of the experimental output. Alternatively, maximal enzyme activities recorded under optimum temperature might be adjusted to growth temperature by applying the Arrhenius equation [54].

**Figure 1:**
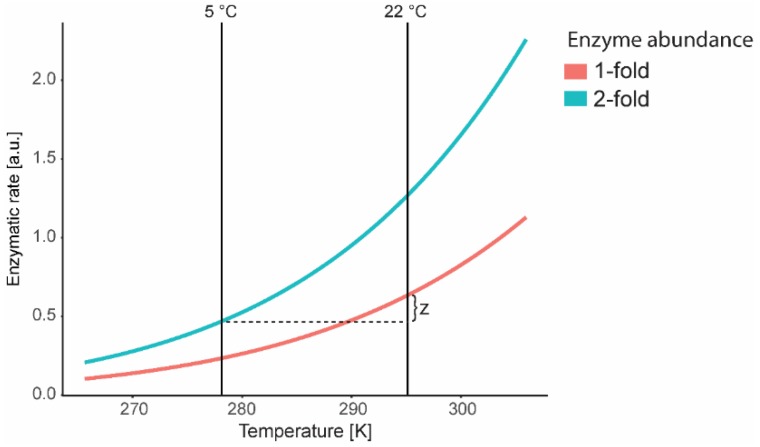
Enzymatic rates with single (1-fold, red, 22 °C) and doubled (2-fold, blue, 5 °C) enzyme abundance. The adjusted v_max_ enzyme activity with doubled abundance at 5 °C is lower than the adjusted activity at 22 °C (factor: z). Enzymatic rates were calculated using the Arrhenius equation (Equation (1)).

Irrespective of the method applied, thermodynamic adjustments need to be considered when enzyme activity and protein amounts are discussed under changing temperature regimes. For example, plants exposed to 5 °C (278.15K) might contain a doubled amount of an enzyme compared to plants exposed to 22 °C (295.15K). Consequently, the doubled enzyme amount results in a doubled maximal reaction rate measured under optimal laboratory conditions (Figure 1).

Nevertheless, the actual maximal enzymatic rate in plants at 5 °C would be lower than in plants exposed to 22 °C by the value of z (difference in enzymatic rate), even though a doubled amount of the investigated enzyme is available (Figure 1). Application of the Arrhenius equation allows the estimation of the actual v_max_ prevalent in the plant under the applied cold condition. Conclusively, cold-induced protein accumulation does not necessarily result in a higher reaction rate in vivo.

## 8. Conclusions

Carbohydrates are central players in plant cold acclimation and future work on the signalling and metabolic regulation involved will extensively broaden our understanding of how they affect and control cold-induced metabolic reprogramming. The combination of findings on subcellular carbohydrate, amino and organic acid metabolism and the dynamics of protein amount and enzyme activities will support our understanding of the initial and long-term stress responses as well as acclimation processes. Thermodynamics need to be considered for a physiologically meaningful interpretation of enzyme kinetics and pathway regulation. Finally, a combination of experimental and mathematical strategies that reveal the role of carbohydrates in cold-induced dynamics at the interface between plant primary and secondary metabolism represents an important topic for future studies on plant-environment interactions.
